# Proteome-wide Mendelian randomisation identifies causal links between blood proteins and myopia

**DOI:** 10.7189/jogh.16.04003

**Published:** 2026-02-20

**Authors:** Fanye Wu, Yuehong Zhou, Xinyu Ma, Zhiyuan Zhao, Shaoyu Wang, Kedi Ma, Siyu Yang, Mingzhe Cao, Guoguo Yi, Min Fu

**Affiliations:** 1 The Second Clinical Medicine School, Southern Medical University, Guangzhou, China; 2 Department of Ophthalmology, Zhujiang Hospital, Southern Medical University, Guangzhou, China; 3 Zhujiang Hospital, Southern Medical University, Guangzhou, Guangdong, China; 4 Department of Ophthalmology, The Seventh Affiliated Hospital of Sun Yat-Sen University, Shenzhen, Guangdong, China; 5 Department of Ophthalmology, The Sixth Affiliated Hospital of Sun Yat-sen University, Guangzhou, Guangdong, China

## Abstract

**Background:**

Myopia is one of the most prevalent eye diseases worldwide, and its incidence is increasing. However, effective pharmaceutical treatments remain limited. We aimed to identify blood proteins causally associated with myopia as potential drug targets.

**Methods:**

We performed a genome-wide association study (GWAS) meta-analysis involving 43 862 myopia cases and 84 820 controls. Then, we conducted a Mendelian randomisation (MR) analysis of blood proteins by utilising the deCODE and UK Biobank Pharma Proteomics Project datasets, and validated the correlations between these characteristics through a cross-sectional study of 50 586 individuals, including 3108 with myopia. Subsequently, through protein-protein interaction (PPI) analyses, we explored potential connections between proteins and existing myopia treatments.

**Results:**

The GWAS meta-analysis found 26 genetic risk loci for myopia, including nine novel loci. The cross-sectional study showed correlations between height, smoking, and myopia. Proteome-wide MR analysis identified 164 plasma proteins potentially causally linked to myopia, with 20 proteins validated in both datasets. Genetic colocalisation analysis, PPI, and drug target analyses identified promising therapeutic targets for myopia.

**Conclusions:**

We identified genetic loci associated with myopia and proteins with potential causal roles in its development. These results indicate new genetic architectures underlying myopia, offering potential treatment targets and a foundation for personalised therapeutic strategies.

Myopia, also known as nearsightedness or shortsightedness, is a refractive error where light that enters the eye parallel to the optic axis focusses in front of the retina during relaxed vision. It ranks among the most prevalent eye diseases globally [1–3], with projections suggesting that it will affect approximately 50% of the world population by 2050, with 10% suffering from high myopia, specifically [4]. While corrective measures like lenses and surgery exist, myopia increases the risk of vision-threatening complications, including myopic macular degeneration, early cataracts, retinal detachment, and glaucoma [5]. Despite its growing prevalence, effective pharmacological interventions remain limited.

Myopia is a multifactorial disease impacted by both environmental and genetic factors [6]. While genome-wide association studies (GWAS) have identified 336 genetic loci associated with myopia, translating these associations into biological mechanisms and therapeutic targets remains challenging [7]. Growing evidence implicates circulating proteins, including hypoxia inducible factor 1 subunit alpha, Wnt Family member 7B, apolipoprotein A1, and leucine rich repeat containing 46, in the pathogenesis of myopia [8–13], yet these relationships are primarily correlative, rather than causal in nature.

To bridge this gap, we employed Mendelian randomisation (MR) to systematically investigate the causal relationships between plasma proteins and myopia. Specifically, we conducted a GWAS meta-analysis using data from the UK Biobank and FinnGen, analysing a total sample size of 43 862 myopia cases and 848 240 controls to identify genetic risk loci associated with myopia. We further performed a proteome-wide (PW) MR study to detect blood proteins associated with myopia.

## METHODS

### GWAS meta-analysis

We performed a fixed-effects GWAS meta-analysis of three GWAS studies: the FinnGenR10 consortium [14] (4106 cases and 429 209 controls) and the UK Biobank (two GWAS studies; 36 623 cases and 41 9031 controls, 3133 cases and 26 184 controls), using the ‘METAL’ package in *R*. We used fastGWA generalised linear mixed model (GLMM), a fast genome-wide association tool based on GLMM, for inflation in test statistics due to the control imbalance of control cases [14]. To account for heterogeneity, we also applied a random-effects model [15]. Our analysis included 9 739 172 single nucleotide polymorphisms (SNPs) with allele frequencies of ≥0.01. We generated a quantile-quantile plot using the ‘qqman’ package in *R*. The SNP-based heritability and linkage disequilibrium (LD) score regression intercept were computed using LD Score (LDSC) software [16]. The genomic inflation factor (λ) was calculated to assess the potential inflation of the test statistic. We used the ‘gassocplot’ in *R* to plot regional association plots for the top SNP at each identified genome-wide significant locus.

### Cross-phenotype genetic correlation of myopia

We investigated the GWAS for myopia for genetic correlation with other traits using the interactive Cross-Phenotype Analysis of GWAS database (iCPAGdb) [17], which presents signals of pairwise traits and shared signals derived from trait associations with LD proxy SNPs. iCPAGdb provides enrichment and similarity metrics using ancestry LD-specific association data by integrating genetic data across >3700 traits from the National Human Genome Research Institute – European Bioinformatics Institute GWAS catalogue. Its output data includes Fisher’s exact test adjusted for a 5% false discovery rate (FDR), Bonferroni’s correction, and Jaccard’s, Sorensen’s, and ChaoSorensen similarity indices. Subsequently, we used LDSC to calculate the genetic correlation between myopia and 12 traits either included in or akin to those analysed by iCPAGdb. We retained only SNPs with a minor allele frequency exceeding 1% and computed genetic correlations based on the default 1000G LD reference data set for Europeans provided by LDSC. Subsequently, we perfomed a series of inverse-variance weighted MR analyses, utilising exclusively genome-wide significant (*P* ≤ 5 × 10^−8^) and independent (*R*^2^ < 0.1) SNPs, in order to evaluate whether these traits could be causally linked with myopia and *vice versa*.

### Cross-sectional study

We obtained data for the cross-sectional segment of this study from the Third Xiangya Hospital of Central South University from 2020 to 2021. All individuals presenting for routine health examinations during this period (n = 103 649) were consecutively recruited to ensure the full inclusion of the study population. At baseline, they filled out a national standardised health screening questionnaire used by the Chinese Health Screening Center, which collects basic demographic information, lifestyle factors, psychological status, personal medical history, and family medical history. 

After excluding duplicates, blanks, and abnormal data (n = 50 586), we retained 52 963 subjects for analysis, of whom 3108 had myopia. Our variables of interest included height, myopia (yes/no), diabetes (yes/no), the number of cigarettes smoked daily (<10, 10–20, 20–30, >30), and smoking duration (<5, 5–10, 10–20, and >20 years) through standardised self-administered questionnaires.

We assessed the association between height, fasting glucose, and myopia using the point-biserial analysis; the relationship between diabetes and myopia using the χ^2^ test; and the links between myopia and both smoking quantity and duration using ordered logistic regression. We used multivariate models mutually adjusted for height, smoking status, diabetes, age, and sex to account for potential confounding between these factors.

### Proteome-wide Mendelian randomisation study and sensitivity analysis

Genetic associations of 4907 proteins in the deCODE plasma proteome were derived from deCODE Genetics’ large-scale protein quantitative trait loci (pQTL) of 35 559 Icelanders. Transient nonspecific interactions were prevented using the SomaScan platform, utilising surface-bound enrichment of proteins and generalised polyanionic competitors [18]. SomaScan v4 (SomaLogic Company, Boulder, Colorado, USA) consists of 4907 aptamer-based assays targeting 4719 proteins. Protein quantitative trait loci for these proteins were generated by genome-wide association testing using 4907 aptamer levels adjusted for age, sex, and sample age as phenotypes and 27.2 million input variants as genotypes. We performed proteome-wide MR (PW-MR) studies using 4907 aptamers.

Plasma pQTL extracted from the UK Biobank Pharma Proteomics Project (UKB-PPP) database were derived from plasma proteomic profiles of 54 219 UK Biobank participants. The UK Biobank-PPP provided pQTLs for 2940 proteins in 54 219 participants [19]. We filtered cis-pQTLs (within ±1 Mb of the gene) and trans-pQTLs (distant) based on specific criteria (minor allele frequency ≥1%, *P* ≤ 5 × 10^−8^, LD *R*^2^<0.1).

We performed MR using significant SNPs *via* the ‘TwoSampleMR’ package in *R*, while applying a FDR correction (5%) [20]. We performed sensitivity analyses, including MR-Egger regression and the Egger intercept test, to assess pleiotropy, evaluated heterogeneity among SNPs using the Cochran’s Q statistic, and employed fixed-effects models for analyses with low heterogeneity (*P* > 0.05) and random-effects models when substantial heterogeneity was detected (*P*  ≤ .05). We also used Steiger directionality testing to assess causality and pleiotropy, where a Steiger *P*-value <0.05 indicated that the explanatory variance of the instrumental variable for the exposure variable was significantly greater than that for the outcome variable, suggesting no reverse effect was detected.

### Bayesian colocalisation analysis

To distinguish shared genetic mechanisms from coincidental linkage, we performed Bayesian colocalisation analysis on all conditionally independent exposure and outcome signals by testing five competing hypotheses:

– H0: there will be no causal variant for either trait; 

– H1: a causal variant affects only the exposure (protein level);

– H2: a causal variant affects only the outcome (myopia);

– H3: distinct causal variants independently affect exposure and outcome;

– H4: a single causal variant influences both traits.

We executed colocalisation assessments of the identified causal proteins utilising default parameters (p1 = 1 × 10^−4^; p2 = 1 × 10^−4^; p12 = 1 × 10^−5^). A higher posterior probability for hypothesis 3 indicated the presence of two autonomous causal SNPs, each associated with a distinct trait [21].

We defined a posterior probability for hypothesis 4 (PPH4) ≥0.75 as strong evidence for colocalisation, indicating that the genetic association with myopia likely arises from the same causal variant influencing protein expression, rather than separate variants in linkage disequilibrium. This significantly strengthens the causal inference from MR analyses by reducing the possibility of coincidental linkage.

We only used SNPs with minor allele frequencies >1% and located within 500 kilobyte of the target gene's gene body. Finally, we calculated the F-statistic, PW-MR, sensitivity, and colocalisation analyses using the Cragg-Donald F-statistic formula for instrumental strengths based on the European 1000G LD reference panel.

To further investigate shared genetic aetiologies among multiple traits, we performed multi-trait colocalisation analysis using hypothesis prioritization colocalization (HyPrColoc). This allowed us to identify regions where a single causal variant might be shared across more than two traits simultaneously.

### PPI and drug targets analysis

We performed drug target analyses using the OpenTargets21 publicly available data, version 24.03 [22], selecting all drugs with evidence of association with the protein of interest. To explore interactions between potential therapeutic targets and myopia, we performed PPI analyses involving identified plasma proteins and previously identified drug therapeutic targets based on data from the STRING database [23] and Cytoscape software [24,25].

## RESULTS

### GWAS meta-analysis of myopia

**Figure 1:**
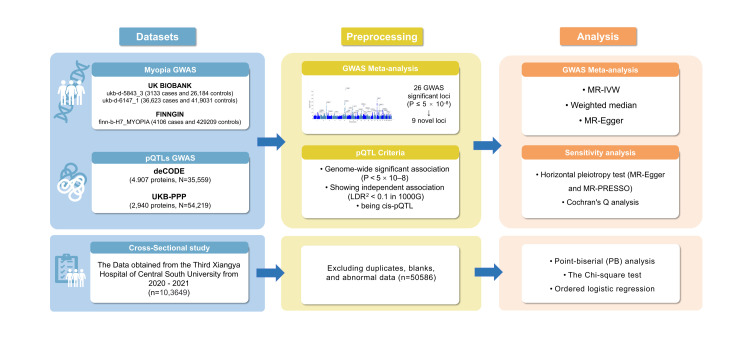
Study flowchart.

We identified 969 significant SNPs (*P* ≤ 5 × 10^−8^) and 26 genetic risk loci with at least one SNP exceeding the genome-wide significance threshold (*P* ≤ 5 × 10^−8^), of which nine were potentially novel susceptibility loci: C4BP4, NT5DC1, NCOA2, COPS7A, ORMDL2, PCCA, CDKN3, ANKFN1, and SPAG4 (**Figure 1**; Figure S1 and Table S1 in the **Online Supplementary Document**).

The genomic inflation factor (lambda) was 1.071. The LD score regression intercept was 0.9319 (SE ¼ 0.0078), and the SNP-based heritability (h2) was 0.0027 (SE ¼ 0.0002), suggesting no genomic inflation. These values were obtained using LDSC.

### Cross-trait and genetic correlation analysis of myopia

We performed cross-phenotype genetic association analyses for myopia genome-wide significant SNPs (*P* ≤ 5 × 10^−8^) using the iCPAGdb. After correcting for the FDR, the cross-phenotype seven traits were still significantly associated with myopia (Table S2 in the **Online Supplementary Document**). The highest enrichments were for optic disc size measurement (*P* = 2.89 × 10^−15^), hypermetropia (*P* = 4.28 × 10^−7^), macula measurement (*P* = 4.88 × 10^−6^), strabismus (*P* = 0.00803), astigmatism (*P* = 0.0349), body height (*P* = 0.0349), and corneal astigmatism (*P* = 0.0385).

We used genetic correlation analyses using GWAS summary statistics for 12 traits and the LDSC software to calculate the SNP-based heritability of each trait and the genetic correlation with myopia (Figure S2 in the **Online Supplementary Document**). After correcting for the FDR (Table S3.1 in the **Online Supplementary Document**), five traits passed the threshold: strabismus (*ρ* = −0.4263; *P* = 8.61 × 10^−6^), astigmatism (*ρ* = 0.488; *P* = 2.03 × 10^−4^), height (*ρ* = 0.091; *P* = 2.03 × 10^−4^), smoking initiation (*ρ* = 0.185; *P* = 1.57 × 10^−10^), and years of education (*ρ* = 0.431; *P* = 3.55 × 10^−60^).

After correcting for the FDR, the two-sample MR showed a correlation of height (*β* = 0.0682; *P* = 4.197 × 10^−16^) and smoking initiation (*β* = 0.272; *P* = 0.0305) with myopia (Table S3.2 in the **Online Supplementary Document**).

### Associations of myopia with height, fasting glucose, and diabetes mellitus

Out of 53 348 participants, 3108 had self-reported myopia. Point-biserial analysis height was positively correlated with myopia (r_point biserial_ = 0.0138; *P* = 0.00139). The χ^2^ test shows positive correlation between smoking and myopia (χ^2^ = 66.068; *P* = 4.356 × 10^−16^).

Among the 11 945 smokers, those who smoked <20 cigarettes per day and smoked for >10 years were less likely to have myopia compared with those who smoked >30 cigarettes per day and smoked for >20 years (*P*  = 1.45 × 10^−5^).

### Proteome-wide Mendelian randomisation studies of myopia

**Figure 2:**
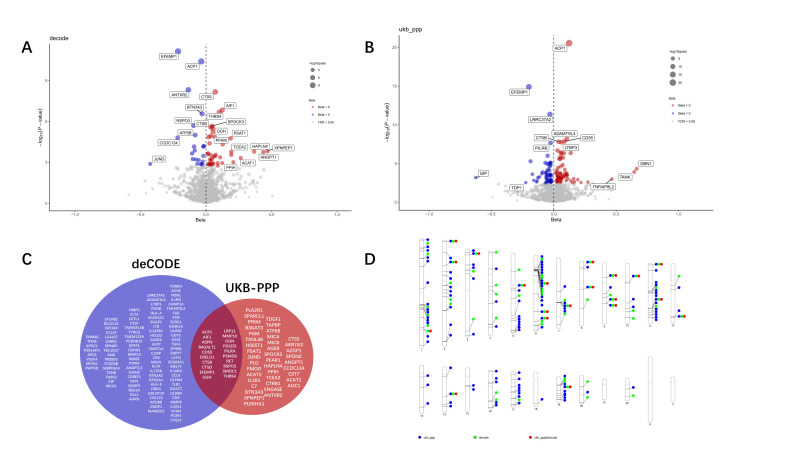
PW-MR studies of myopia. Volcano plot of PW-MR study using (**Panel A**) deCODE and (**Panel B**) UKB-PPP, displaying the strength (−log_10_ *P*-value) *vs*. magnitude (beta coefficient) of protein-myopia associations. Annotated proteins passed the 5% FDR inverse-variance weighted *P*-value threshold. The blue and red colours represent a negative and positive effect, respectively. **Panel C.** Venn diagram depicting proteins associated with myopia in deCODE only, UKB-PPP only, or both. **Panel D.** PhenoGram of GWAS meta-analysis and PW-MR study significant associations. The blue dots represent the top genome-wide significant loci, the green dots represent the PW-MR significant proteins, and the red dot represents both simultaneously.

We investigated the associations of 4907 and 2940 plasma protein levels with myopia using genetic association summary statistics from 35 559 Icelanders in deCODE Genetics and 54 219 Europeans in the UKB-PPP, respectively. After strictly following the screening criteria for instrumental variables, we included 1988 proteins from the deCODE and 1808 from UKB-PPP in the analysis (Tables S4.1 and S4.3 in the **Online Supplementary Document**). One hundred sixty-four plasma proteins had positive causal associations with myopia, based on inverse-variance weighted or Wald ratio results (FDR-corrected *P* < 0.05). A total of 61 proteins were identified in deCODE, and 123 were identified in UKB-PPP (**Figure 2**, Panels A and B). The results of multiple sensitivity analyses, including MR-Egger, weighted median, and contamination mixture, showed little evidence of pleiotropy (Tables S4.1 and S4.3 in the **Online Supplementary Document**). Twenty proteins were verified in two datasets (**Figure 2**, Panel C). The F-statistics of the genetic instruments for each protein showed solid instrument strength (Tables S4.2 and S4.4 in the **Online Supplementary Document**). A PhenoGram representing the chromosomal location of the 164 uniquely identified proteins and that of the susceptibility loci identified by GWAS meta-analysis is depicted (**Figure 2**, Panel D).

We performed gene colocalisation analyses in the upstream and downstream ±1Mb range of their respective genes to explore potential associations with myopia. Among the 164 proteins identified by MR analysis, seven demonstrated strong colocalisation evidence (PPH4 ≥ 0.75) in at least one data set. Acid phosphate 1 (ACP1) showed strong evidence of colocalisation (one causal variant shared between both traits) in deCODE (PPH4 = 0.918) and UKB-PPP (PPH4 = 0.917). This analysis identified one causal variant in deCODE (rs11678766) and one causal variant in Fenland (rs10204657). Angiopoietin-1 (ANGPT1) and XPNPEP in deCODE and EFEMP, DBN1, TAN, and IL12RB1 demonstrated strong colocalisation in UKB-PPP were strongly colocalised with myopia. The HyPrColoc-based analysis of associations between myopia, five LDSC-screened characteristics, and 20 protein pQTLs and eQTLs in whole blood showed no evidence for colocalisation of myopia and trait proteins.

The Steiger test indicated no evidence of reverse causal association for the MR analysis (Tables S4.6 and S4.7 in the **Online Supplementary Document**), suggesting that the 164 proteins may represent new blood proteins that are causal drivers of myopia, rather than its consequence.

### PPI reveals association of potential drug targets with current myopia drug targets

**Figure 3:**
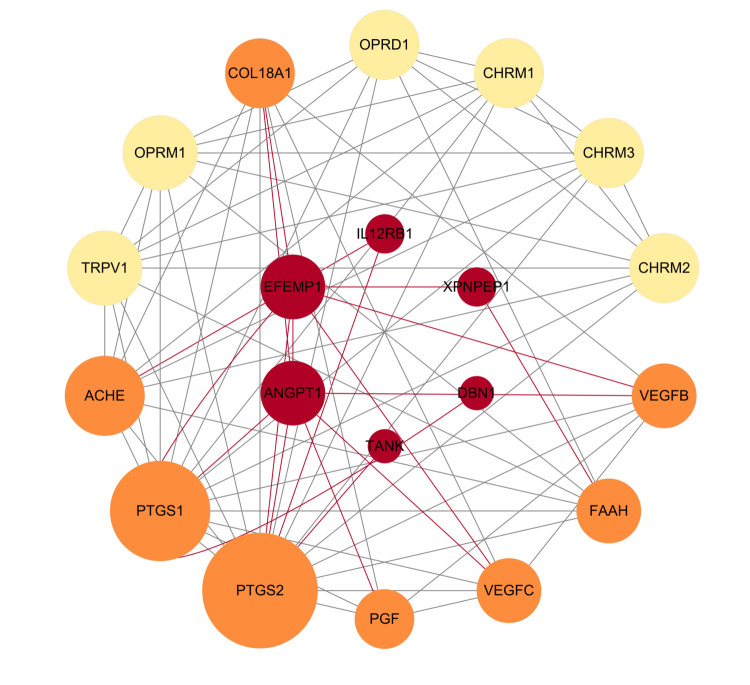
Protein-protein interaction network between myopia's causative proteins and current drug targets. Red circles represent plasma proteins. Orange solid circles indicate current myopia drug targets associated with potential proteins, while yellow solid circles indicate current myopia drug targets without such associations. The size of the circles indicates the number of interacting proteins. ACHE – acetylcholinesterase, ANGPT1 – angiopoietin 1, CHRM1 – cholinergic receptor muscarinic 1, CHRM2 – cholinergic receptor muscarinic 2, CHRM3 – cholinergic receptor muscarinic 3, COL18A1 – collagen type XVIII alpha 1 chain, DBN1 – drebrin 1, EFEMP1 – EGF containing fibulin extracellular matrix protein 1, FAAH – fatty acid amide hydrolase, IL12RB1 – interleukin 12 receptor subunit beta 1, OPRD1 – opioid receptor delta 1, OPRM1 – opioid receptor mu 1, PGF – placental growth factor, PTGS1 – prostaglandin-endoperoxide synthase 1 (COX-1), PTGS2 – Prostaglandin-endoperoxide synthase 2 (COX-2), TANK – TRAF family member associated NFKB activator, TRPV1 – transient receptor potential cation channel subfamily V member 1, VEGFB – vascular endothelial growth factor B, VEGFC – vascular endothelial growth factor C, XPNPEP1 – X-prolyl aminopeptidase 1

The PPI network revealed six identified proteins (ANGPT1, XPNPEP1, EFEMP1, DBN1, TANK, IL12RB1) that previously interact with 14 current drug targets (**Figure 3**). Specifically, PTGS2 and PTGS1 are both targets of ketorolac, ketorolac tromethamine, nepafenac, and acetaminophen. ACHE is a target of echothiophate and echothiophate iodide. VEGFB is a target of conbercept. FAAH is a target of acetaminophen, and VEGFC is a target of conbercept. Notably, ACP1 does not interact with any existing drug targets.

## DISCUSSION

To better understand the genetic structure of myopia, we conducted a meta-analysis of 43 862 individuals with myopia and 848 240 individuals without myopia at UK Biobank and FinnGen. Seventeen of 26 genetic risk loci in our analysis emerged as risk loci for myopia or associated with myopia.

5′-nucleotidase domain-containing 1(NT5DC1) is a member of the 5′(3′) - deoxygenated nucleotide family and has been linked to attention-deficit hyperactivity disorder (ADHD), bipolar disorder, Alzheimer’s disease, and other psychiatric disorders [26,27]. For example, ADHD patients, particularly those receiving no treatment, were more likely to develop myopia than non-ADHD patients [28,29]. This could be attributed to their higher susceptibility to excessive smartphone and Internet usage increasing their likelihood of developing myopia [30,31].

Here we calculated the cross-phenotype of myopia using iCPAGdb and validated it with LDSC and MR. In our analysis, we identified a significant positive genetic correlation between myopia and years of education (*ρ* = 0.431; *P* = 3.55 × 10^−60^). This finding aligns with the well-established understanding that increased years of schooling are a risk factor for myopia. [32]. Furthermore, with higher levels of education, there is a significant rise in the occurrence of myopia, suggesting that the duration of education may have a cumulative impact [33]. MR provides genetic support for it [34], which aligns with our conclusion.

Exposure to second-hand smoke was related to high-altitude myopia and the earlier onset of myopia [35]. Possible mechanisms involve stimulating nicotine receptors in the retina and other eye tissues [36,37]. Notably, we noted a strong association between body height and myopia in our MR, LDSC, and cross-sectional analyses. Research on newborns, children, and adults has demonstrated a relationship between body shape and axial length [38–41]. The shared factors between myopia and height may originate from various sources. Chondrocytes and scleral fibroblasts share overlapping genetic expression patterns, specifically in genes such as Indian hedgehog, type X collagen, and MMP13 [42]. Research has demonstrated that patients with primary growth hormone receptor resistance who get IGF-1 treatment exhibit increased axial lengths and elevated corneal curvature compared to patients with [43]. Intravitreal injection of IGF-1 in chicks significantly increases ocular elongation and myopia [44]. Bone morphogenetic protein-2 can induce the directed differentiation and proliferation of undifferentiated mesenchymal stem cells into chondrocytes and osteoblasts [45], and was significantly down-regulated in the retinal pigment epithelium of guineas pig after contact lenses induced myopia [46]. These findings highlight the necessity for further investigation into growth-related pathways in myopia pathogenesis.

We identified cis-acting pQTL for thousands of proteins in the deCODE and UKB-PPP datasets, where 164 proteins were associated with myopia after correcting for the FDR, and where seven passed colocalisation. The PW-MR and bidirectional analyses deepened our understanding of myopia biomarkers and pathophysiology. ACP1 exhibited a strong association with myopia in both databases. Retinal ACP1 expression was up-regulated in guinea pigs with lens-induced myopia compared to controls [47]. Variants in ACP1, which encodes low-molecular-weight protein tyrosine phosphatase, affect serum glucose concentration and insulin sensitivity in humans by dephosphorylating the insulin receptor [48–51]. The observed correlation between myopia and type 2 diabetes suggests shared pathophysiological pathways involving insulin resistance [52,53]. Studies in chick eyes demonstrate that insulin and IGF-1 receptors are expressed in the sclera, choroid, and retina [54,55]. Intravitreal insulin and IGF-1 injections significantly increased ocular elongation and myopia progression [44]. Interestingly, in PPI analyses, ACP1 showed no association with existing myopia drug targets, suggesting its potential druggability represents a novel therapeutic avenue regarding pharmacological modulation. Low-molecular-weight protein tyrosine phosphatase can be activated by adenine, a mechanism implicated in progressive type 2 diabetic kidney disease [56–58]. While ACP1's absence from known drug-interaction networks presents development challenges, it simultaneously represents a unique therapeutic opportunity targeting a novel mechanism in myopia pathogenesis. These findings position ACP1 as a promising novel target for myopia treatment. EFEMP1, a key scaffolding protein of the extracellular matrix, is expressed in scleral fibroblasts of myopic guinea pigs [59]. It influences collagen and aggregated proteoglycan secretion from scleral cells, critically regulating extracellular matrix remodelling in the sclera [60]. DBN1 acts as a multifunctional cytoskeleton regulator, is phosphorylated by CDK5, and plays a vital role in apical-basal elongation of epithelial cells [61,62]. These effects affect lens morphogenesis and growth. Knockout of DBN1 revealed that the prominent phenotypes found in mouse eyes include a significant reduction in eye and lens size [63]. ANGPT1 is expressed in the choroid and regulates choroidal capillary development and vortex vein patterns [64–66]. Given that choroidal neovascularisation is a vision-threatening complication of high myopia, ANGPT1’s role in vascular homeostasis may hold therapeutic relevance [67]. Our identification of proteins involved in insulin/IGF-1 signalling (ACP1), extracellular matrix organisation (EFEMP1, DBN1), and vascular regulation (ANGPT1) converges on known biological pathways in myopia pathogenesis. Specifically, the height-myopia relationship may be mediated through IGF-1 signalling pathways that simultaneously influence skeletal growth and scleral remodelling, with our protein findings representing measurable circulating components of these shared processes.

Our study highlighted significant potential of multiple plasma proteins for clinical translation, which may serve as biomarkers for early screening in paediatric populations, where a multi-protein panel could offer superior predictive accuracy compared to single biomarkers [68]. Several candidates also interact with established drug targets (*e.g.* PTGS1, VEGFC), suggesting immediate opportunities for drug repurposing, such as developing ACP1 inhibitors or ANGPT1-TIE2 modulators. To advance these findings, we propose a multi-tiered strategy including functional validation in animal models, longitudinal clinical studies in children, randomised trials of repurposed drugs, and multi-ethnic proteomic analyses to ensure global applicability.

Several limitations of our study need to be recognised. First, the pQTL and GWAS data were derived exclusively from European populations, which might compromise the generalisability of our findings to other racial or ethnic groups. Second, caution is needed in interpreting PPH4, as its low values not indicate a lack of evidence supporting colocalisation, especially when posterior probability for hypothesis 3 is also reduced due to limited statistical efficacy [69]. Third, the cross-sectional data from hospital cohorts may have introduced selection bias, as participants with more severe myopia or comorbid conditions may have been overrepresented. Simultaneously, our reliance on self-reported myopia status could lead to outcome misclassification, particularly for mild cases. Fourth, further mechanistic studies are needed to elucidate the biological pathways underlying our *in vivo* and *in vitro* findings, particularly regarding the therapeutic potential of the identified proteins.

We identified novel biomarkers of myopia and improved our understanding of its pathogenesis. Our LDSC and cross-sectional exploration revealed traits associated with myopic ducts, while the MR and colocalisation analyses identified seven plasma proteins related to myopia, among which ACP1 has the potential to be a drug target. These findings offer personalised prevention strategies for individuals at genetic risk of myopia and provide novel directions for targeted therapies.

## CONCLUSIONS

We identified nine novel genetic risk loci through a proteome-wide Mendelian randomisation analysis; 164 plasma proteins were causally associated with myopia, of which 20 were validated across datasets. ACP1 emerged as a causal protein of myopia with potential therapeutic value, suggesting it should be prioritised for entering the preclinical research stage. Further cross-sectional analyses confirmed positive associations between height, smoking, and myopia risk. Our study provides a new direction for analysing the genetic mechanism of myopia and developing targeted therapies.

## Additional material


Online Supplementary Document
