# Supplementary material for: Proteome-wide Mendelian randomisation identifies causal links between blood proteins and myopia
Source: J Glob Health. 2026 Feb 20;16:04003. doi: 10.7189/jogh.16.04003 (PMC12922467; doi:10.7189/jogh.16.04003)
Supplement: Online Supplementary Document [file jogh-16-04003-s001.zip › jogh-16-04003-s001.pdf]

**Supplement to:Wu F, Zhou Y, Ma X, Zhao Z, Wang S, Ma K, Yang S, Cao M, Yi G, Fu M. Proteome-wide Mendelian randomisation identifies causal links between blood proteins and myopia. J Glob Health. 2026;16:04003.**

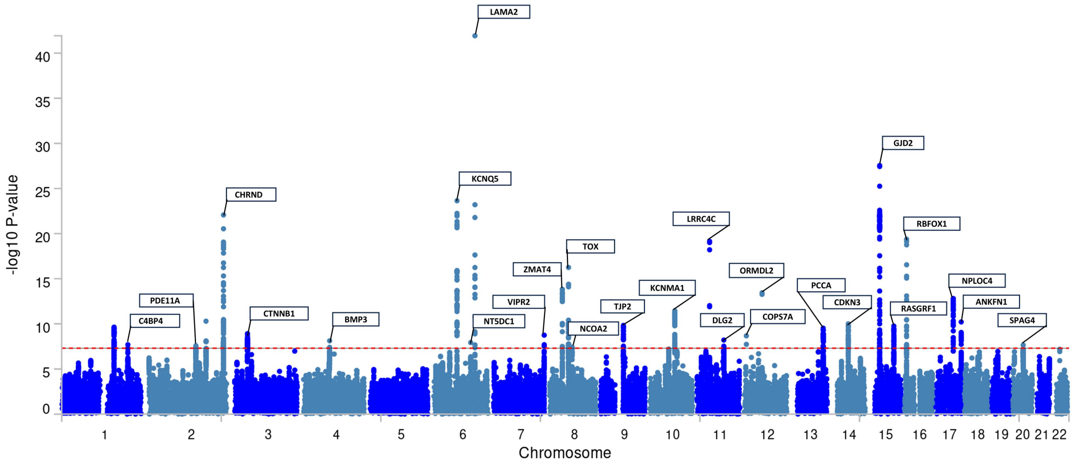


**Figure S1.** Manhattan plot of myopia GWAS meta-analysis. Genetic loci harboring top SNPs associated with myopia ($P \leq 5 \times 10^{-8}$) are shown. The red dashed line represents the genome-wide significance threshold of $P \leq 5 \times 10^{-8}$.

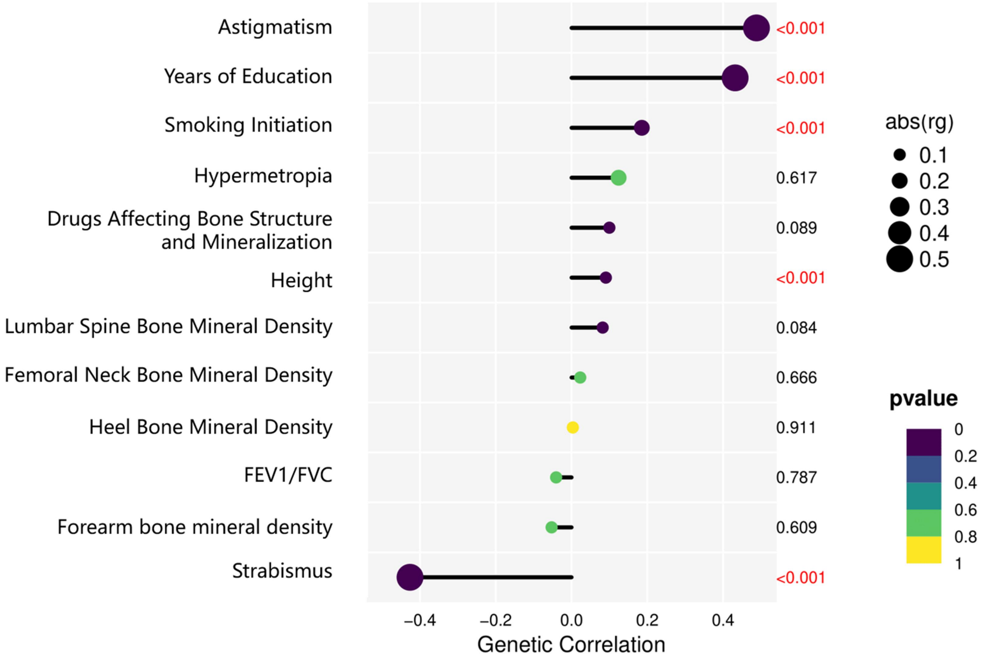


**Figure S2.** Cross-trait analysis and genetic correlation of acute pancreatitis with other traits. The size of the dots reflects the strength of the genetic correlation, and the bottom horizontal coordinate indicates a positive or negative correlation.

**Table S1.** Alignment with JoGH GRABDROP guidelines

| | |
|---|---|
| 1. Please list all papers published by each co-author in previous three years that were based on secondary analysis of a big data repository | Yi G, Li Z, Sun Y, Ma X, Wang Z, Chen J, Cai D, Zhang Z, Chen Z, Wu F, Cao M, Fu M. Integration of multi-omics transcriptome-wide analysis for the identification of novel therapeutic drug targets in diabetic retinopathy. J Transl Med. 2024 Dec 24;22(1):1146. doi: 10.1186/s12967-024-05856-7. PMID: 39719581; PMCID: PMC11667901.<br>Sun Y, Zhang Z, Chen Z, Li Z, Wang Z, Wu F, Ma X, Wang S, Cao M, Yi G, Fu M. Novel High-Resolution Lipidomes Could Serve as New Biomarkers for Diabetic Retinopathy: A Bidirectional and Mediated Mendelian Randomization Study. J Cell Mol Med. 2025 Jun;29(11):e70614. doi: 10.1111/jcmm.70614. PMID: 40464192; PMCID: PMC12134777. |
| 2. Please explain the key elements of your study design and the use of the available datasets that make your study an original scientific contribution | A Multi-layered Research Framework: We constructed a complete logical chain from genes to proteins to phenotypes. First, we conducted a GWAS meta-analysis on over 890,000 samples from FinnGen and UK Biobank; subsequently, we systematically assessed the causal relationships between thousands of plasma proteins and myopia using whole-proteome Mendelian randomization (PW-MR); Finally, we validated the identified associations using real-world clinical data from over 50,000 individuals at Xiangya Hospital.<br>Strategic Application of High-Resolution Datasets: By cross-referencing two independent, large-scale proteomics databases—deCODE and UKB-PPP—we significantly enhanced result reliability, successfully identifying 20 high-confidence disease-associated proteins validated in both databases.<br>Uncovering novel scientific discoveries: Our research not only identified 26 genetic risk loci—including 9 previously unreported novel loci—but also pinpointed 164 plasma proteins causally linked to myopia.<br>Clear Clinical Translation Implications: We have for the first time identified the ACP1 protein as a novel therapeutic target with high potential for myopia treatment. Furthermore, we revealed genetic associations between myopia and clinical characteristics such as height and smoking, proposing key biological pathways like IGF-1 as mediators. This provides scientific evidence for personalized prevention and precision treatment of myopia.<br>By integrating large-scale genetic data, proteomics analysis, and clinical validation, our research bridges the gap from simple correlation observation to in-depth exploration of causal mechanisms. |
| 3. Please list all publications that addressed similar research questions in the same dataset and indicate where you cited them in your paper | Hysi, P.G., Choquet, H., Khawaja, A.P. et al. Meta-analysis of 542,934 subjects of European ancestry identifies new genes and mechanisms predisposing to refractive error and myopia. Nat Genet 52, 401–407 (2020). https://doi.org/10.1038/s41588-020-0599-0<br>**We cited this paper in the Introduction section to illustrate that although GWAS has identified 336 myopia-associated loci, translating them into therapeutic targets remains challenging; and referenced it in the Discussion section.**<br>Mountjoy E, Davies NM, Plotnikov D, Smith GD, Rodriguez S, Williams CE, Guggenheim JA, Atan D. Education and myopia: assessing the direction of causality by mendelian randomisation. BMJ. 2018 Jun 6;361:k2022. doi: 10.1136/bmj.k2022. Erratum in: BMJ. 2018 Jul 4;362:k2932. doi: 10.1136/bmj.k2932.<br>**We cite this article in the Discussion section to support our research conclusion that genetic evidence supports increased years of schooling as a causal risk factor for myopia.**<br>Sun, B.B., Chiou, J., Traylor, M. et al. Plasma proteomic associations with genetics and health in the UK Biobank. Nature 622, 329–338 (2023). https://doi.org/10.1038/s41586-023-06592-6 |

| | |
|---|---|
| | **We cite this article in the Methods section as the source description for the UKB-PPP protein quantitative trait locus (pQTL) data we used.** |
| 4. Please explain how you addressed multiple testing through an appropriately rigorous statistical threshold and indicate this in the methods section | We addressed the potential risk of false positives from multiple testing by implementing rigorous statistical thresholds across all stages of our analysis. For the initial GWAS meta-analysis, we adopted the genome-wide significance threshold of $P \leq 5\times10^{-8}$ to identify genetic risk loci. To account for multiple comparisons in our cross-phenotype and genetic correlation analyses, we applied a 5% false discovery rate (FDR) correction.<br>Similarly, for the proteome-wide Mendelian randomization, we utilized the TwoSampleMR package with a 5% FDR adjustment, considering only proteins with $P_{fdr} < 0.05$ as significant. Finally, to ensure the robustness of our causal inferences and distinguish shared genetic mechanisms from coincidental linkage, we performed Bayesian colocalization analysis, setting a stringent posterior probability threshold of PPH4 ≥ 0.75 as strong evidence for colocalization. These thresholds are explicitly detailed in the respective subsections of our Methods section. |
| 5. Please declare to what extent have AI chatbots been used in developing your paper and to which parts of the paper did they contribute | We declare that AI chatbots were used in the development of this manuscript strictly for the purposes of linguistic refinement, including grammar correction and language polishing. This assistance was sought to ensure the clarity and professional tone of the English-language presentation, particularly within the Abstract, Introduction, and Discussion sections. |
